# Retraction: Failure of an Ancient Breast Implant Can Lead to Significant Morbidity

**DOI:** 10.7759/cureus.r30

**Published:** 2021-04-15

**Authors:** Ali K Mohammed, Sherif Monib

**Affiliations:** 1 Surgery, Watford General Hospital, Watford, GBR; 2 Breast Surgery, St. Albans and Watford General Hospitals, West Hertfordshire Hospitals NHS Trust, London, GBR

This article has been retracted and removed due to recently discovered issues with the patient consent process. Unbeknownst to the authors at the time patient consent was granted, the patient's next of kin had power-of-attorney and therefore should have been consulted regarding patient consent. As a result, the patient consent form is not valid and the patient now wishes for the article to be removed. The authors have formally requested this retraction.

